# Kinematic Validation of Postural Sway Measured by Biodex Biosway (Force Plate) and SWAY Balance (Accelerometer) Technology

**DOI:** 10.1155/2019/8185710

**Published:** 2019-12-21

**Authors:** Birendra M. Dewan, C. Roger James, Neeraj A. Kumar, Steven F. Sawyer

**Affiliations:** Department of Rehabilitation Sciences and the Center for Rehabilitation Research, School of Health Professions, Texas Tech University Health Sciences Center, Lubbock, TX 79430, USA

## Abstract

**Background:**

The Biodex Biosway® Balance System and SWAY Balance® Mobile smartphone application (SBMA) are portable instruments that assess balance function with force plate and accelerometer technology, respectively. The validity of these indirect clinical measures of postural sway merits investigation.

**Purpose:**

The purpose of this study was to investigate the concurrent validity of standing postural sway measurements by using the portable Biosway and SBMA systems with kinematic measurements of the whole body Center of Mass (COM) derived from a gold-standard reference, a motion capture system.

**Study Design:**

Cross-sectional; repeated measures.

**Methods:**

Forty healthy young adults (21 female, 19 male) participated in this study. Participants performed 10 standing balance tasks that included combinations of standing on one or two legs, with eyes open or closed, on a firm surface or foam surface and voluntary rhythmic sway. Postural sway was measured simultaneously from SBMA, Biosway, and the motion capture system. The linear relationships between the measurements were analyzed.

**Results:**

Significant correlations were found between Biosway and COM velocity for both progressively challenging single and double leg stances (*τ*_b_ = 0.3 to 0.5, *p* < 0.01 to <0.0001). SBMA scores and COM velocity were significantly correlated only for single leg stances (*τ*_b_ = −0.5 to −0.6, *p* < 0.0001). SBMA scores had near-maximal values with zero to near-zero variance in double leg stances, indicating a ceiling effect.

**Conclusion:**

The force plate-based Biodex Biosway is valid for assessing standing postural sway for a wide range of test conditions and challenges to standing balance, whereas an accelerometer-based SWAY Balance smartphone application is valid for assessing postural sway in progressively challenging single leg stance but is not sensitive enough to detect lower-magnitude postural sway changes in progressively challenging double leg stances.

## 1. Introduction

Postural sway is an important metric that provides information about balance function, postural control, and risks of falls. Increased postural sway is an overall expression of the derangement of the postural control mechanisms [1, 2]. Quantitative posturography provides an objective measurement of postural sway. It usually includes a set of standing balance test protocols and recording of postural sway with an objective instrumentation under controlled conditions [3]. The 3-dimensional (3D) motion capture system (MOCAP) is considered a gold-standard reference for the assessment of postural stability which offers a high level of accuracy and reliability necessary to record small motions [4–6]. However, it requires substantial cost, space, time, and trained personnel for use. Clinicians without access to a motion capture system or clinicians who are in the field need valid alternatives that can objectively quantify postural sway. Portable quantitative posturography can provide clinicians with on-the-go easy access to objective balance assessment. The Biodex Biosway Portable Balance System (Biodex Medical System, Shirley, NY) is a reliable quantitative posturography system that measures postural sway indirectly using force platform technology to detect changes in center of pressure (COP) [7–10]. It assesses balance using the modified Clinical Test of Sensory Integration and Balance (mCTSIB) protocol [10] (see Methods).

The use of accelerometers is increasing in the assessment of gait and postural sway [11, 12]. Accelerometers are a low-cost, commercially available, and portable system of measuring variability of movement and balance [13] that have been found to differentiate the gait in older adults with and without stability problems [14] and in older adults who are frequent fallers [15]. SWAY Balance™ mobile application (SBMA) (version 2.1.1, SWAY Medical, Tulsa, OK) is a smartphone application that purports to provide objective measurement of thoracic sway by using integrated triaxial accelerometers to measure motion of the device during performance of a series of balance tasks [16]. The phone with the SBMA is normally held against the chest at midsternum level by the subject using both hands during the testing protocol. The SBMA has been shown to have excellent overall reliability [17].

SBMA measures movement of the thorax, whereas Biosway measures COP with force plates embedded in a foot platform. Therefore, the two instruments differ in their indirect measurement of postural sway. One study found that lower extremity (hamstring and quadriceps) strength did not have an effect on standing balance as measured by SBMA [18]. In contrast, the COP depends primarily on motor control of lower extremity and ankle muscles [19]. Furthermore, the two instruments use different algorithms to compute their balance measures (see Methods). These differences make it difficult to interpret and compare the results of balance assessment tests from the two instruments. This necessitates validation of the outcome measures from the two instruments with a concurrent measurement from an objective direct measure of postural sway.

**Figure 1:**
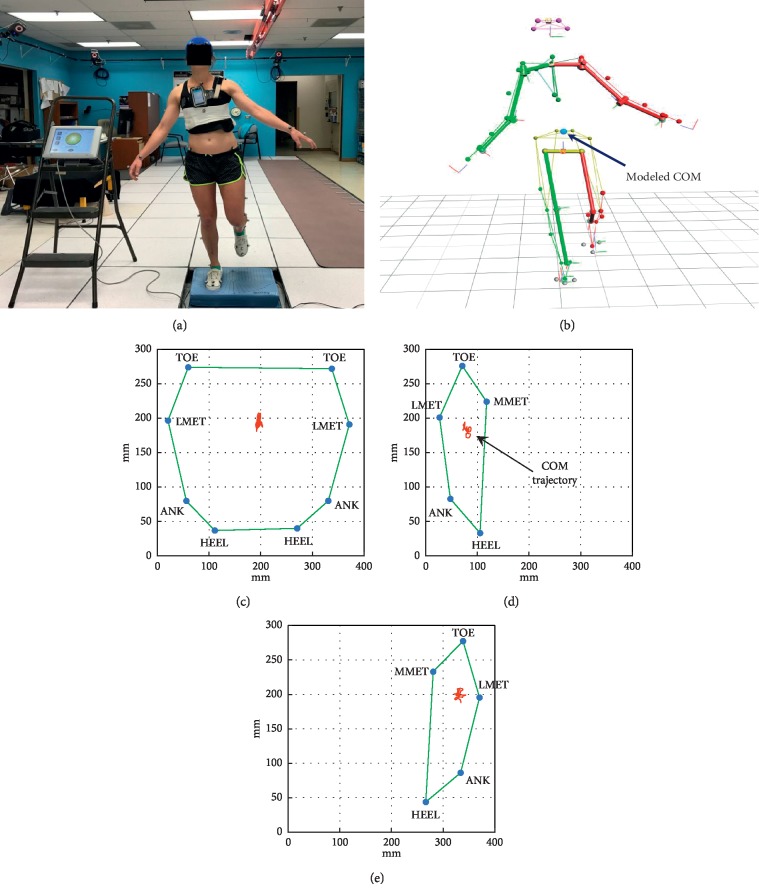
Experimental setup. (a) The reflective markers were attached to each subject according to Plug-in Gait (PiG). The iPhone with SWAY Balance Mobile Application (SBMA) was secured to the subject's chest, and the subject stood on the Biosway platform or foam pad placed over the platform. For establishing Base of Support (BOS), markers were placed on the foot at tip of second toe (TOE), lateral head of 5^th^ metatarsals (LMET), lateral malleoli (ANK), heel (HEEL), and medial head of 1^st^ metatarsals (MMET). (b) Marker trajectories were recorded through the motion capture system (MOCAP) to generate a kinematic stick figure and process a modeled Center of Mass (COM) position. The trajectory of COM was projected onto BOS for a representative subject during (c) double leg, (d) single leg on left foot, and (e) single leg on right foot.

COM movement provides direct information about postural sway and postural control in relation to balance. Even though COM and COP movements are closely related during quiet standing, COM is a more valid measure of levels of disequilibrium during high velocity movement [1]. COM is the key variable controlled by the central nervous system during postural control [20]. With a 3D MOCAP, the whole-body COM can be mathematically modeled as the weighted average of the COMs of all individual body segments (Figures 1(a) and 1(b)) [21]. Standing balance is an ability to maintain the body's Center of Mass (COM) over its Base of Support (BOS) [22]. The size of BOS affects postural sway during quiet stance [23, 24]. To provide a more accurate representation of balance characteristics across subjects with dynamically changing BOS during different standing stances such as double leg stance vs. single leg stance, BOS area needs to be taken into consideration when evaluating COM excursion. COM, as well as COP, has inherently greater room for posturally stable excursion within a larger BOS, and the maximal posturally stable excursion is inherently limited with a smaller BOS. Therefore, the current study incorporated the measurements of BOS area with the measurements of COM excursion during a variety of standing stances and test protocols for all subjects.

The purpose of this study was to investigate the concurrent validity of the SBMA and Biosway with the direct kinematic measure of postural sway obtained from MOCAP. The study specifically aimed to determine if the outcome measures from Biosway and SBMA correlate in a statistically significant fashion with COM Balance indices and with each other in progressively challenging standing stances and if there are differences in postural sway as measured by the SBMA and Biosway based on difficulty level of conditions. It was hypothesized that the outcome measures from Biosway and SBMA will have significant correlation with COM Balance indices and with each other in progressively challenging standing stances and that there will be significant differences in postural sway as measured by the SBMA and Biosway based on difficulty level of conditions.

## 2. Methods

### 2.1. Sampling and Subjects

A convenience sample of forty subjects (19 males and 21 females, age: 24.6 ± 3.1 years, height: 170.3 ± 79.0 cm, mass: 65.0 ± 11.0 kg, body mass index (BMI): 22.3 ± 2.9 kg·m^−2^) were recruited from the student population of the affiliated university and from the general population by means of posted flyers and word-of-mouth. Signed informed consent approved by the Institutional Review Board for the Protection of Human Subjects at the university was obtained from the subjects before participating in the study. As a screening test, volunteers performed preliminary standing tasks that were identical to the experimental protocol and were included in the study only if they were able to maintain balance in all standing tasks and could complete the protocols without needing to sit. The screening tasks also served as trial familiarization. Other exclusion criteria were any health condition that would affect standing balance, taking any medications that could affect the ability to maintain standing balance for at least 5 minutes, a BMI ≥30, and pregnancy [17, 24]. None of the recruited subjects were excluded from the study.

### 2.2. Instrumentation

Kinematic data were acquired using a 3D MOCAP with eight infrared cameras (T-40s, VICON Motion Systems Ltd., Centennial, CO; 100 Hz). A Plug-in Gait (PiG) Full-Body model with 39 markers was used for processing COM trajectories [25]. Kinematic model reconstruction from markers, joint center calculations, segment definitions, and processing of COM trajectories were achieved using the default PiG Full-Body pipeline in Vicon Nexus. The Marker trajectory data were filtered with a 4^th^ order, two-pass, zero lag Butterworth low pass filter at 6 Hz in Nexus.

The Biosway sampling rate of COP is 20 Hz. The system's calculated outcome measure is called the Sway Index (SI), which is the root mean squared error distance of the COP two-dimensional coordinates (equation (1)) [10]. A larger SI score represents more postural sway.

SBMA quantifies the amount of subject's thoracic movement by measuring jerk (m/s^3^ or g/s), which is the rate of change in acceleration with respect to time [26]. The software samples instantaneous acceleration at 10 Hz from a smartphone accelerometer for each of three directional axes and uses a proprietary algorithm to generate a score on a scale of 0 to 100. A larger SBMA score represents less postural sway.

### 2.3. COM Balance Indices

COM and BOS marker trajectories derived from Nexus were exported to MATLAB (R2016b, MathWorks Inc., Natick, MA) for computation of COM Balance indices and mean BOS area. Mean BOS area was calculated as the average of the area of polygon at each frame joining the BOS markers (Figures 1(c)–1(e)). The trajectory of the COM was mathematically projected onto the BOS to determine the following balance indices.

(1) $$\begin{array}{c} \text{COM sway index}\left(\text{COM SI}\right)\left(\text{m}\right)=\sqrt{\frac{1}{N}\left(\overset{N}{\underset{i=1}{\sum }}\left({\left(x_{i}-x_{m}\right)}^{2}+{\left(y_{i}-y_{m}\right)}^{2}\right)\right)}, \end{array}$$

(2) $$\begin{array}{c} \text{COM mean displacement velocity}\left(\text{COM velocity}\right)\left(\text{m}\cdot {\text{s}}^{-1}\right)=\frac{\sum_{i=1}^{N}\sqrt{{\left(x_{i}-x_{i-1}\right)}^{2}+{\left(y_{i}-y_{i-1}\right)}^{2}}/\left(t_{i}-t_{i-1}\right)}{N}, \end{array}$$

where *N* = number of frames, *x*_*i*_ = location of COM in *x*-axis at frame *i*, *x*_*m*_ = mean COM *x*-coordinate value, *y*_*i*_ = location of COM in *y*-axis at frame *i*, *y*_*m*_ = mean COM *y*-coordinate value, and *t*_*i*_ = time at frame *i*.

To account for the difference in BOS in different stance conditions for each subject, the above balance indices were standardized by dividing each by the mean BOS area.

### 2.4. Procedures and Protocol

Subjects donned athletic shorts and sports top (for females) or t-shirt (for males). Anthropometric measurements (height, weight, shoulder offset, elbow width, wrist width, hand thickness, leg length, knee width, and ankle width) were recorded, and lightweight reflective marker spheres (14 mm) were affixed to the head, thorax, arms, and legs of the subject according to the PiG Full-Body model [25] and BOS marker positions (Figure 1(a)). An iPhone (model: 5c, iOS version: 10.3.1) running the SBMA was attached to a belt and secured around the chest at midsternum level. After positioning the subject's feet on Biosway's platform as instructed by the Biosway software, subjects performed the following 10 standing tasks of the test protocol:

  C1: eyes open, firm surface, double leg stance
  C2: eyes closed, firm surface, double leg stance
  C3: eyes open, foam surface, double leg stance
  C4: eyes closed, foam surface, double leg stance
  C5: eyes open, foam surface, single leg stance—right
  C6: eyes open, foam surface, single leg stance—left
  C7: eyes closed, firm surface, single leg stance—right
  C8: eyes closed, firm surface, single leg stance—left
  C9: eyes open, firm surface, voluntary sway at anterior-posterior direction
  C10: eyes open, firm surface, voluntary sway at medial-lateral direction

A four-inch tall foam pad on the top of the platform was used to create an unstable surface for C3–C6. The order in which all the subjects performed test conditions were C1, C2, C3, C4, C8, C7, C6, C5, C9, and C10. C1–C8 are in the increasing order of difficulty. C5 and C6 as well as C7 and C8 are of the same inherent level of difficulty but performed on either leg. C1–C4 constitute the protocol for mCTSIB, each of 30 seconds in duration. C5–C8 include single leg stances, each of 10 seconds in duration. For C9 and C10, subjects were asked to voluntarily sway for 10 seconds at a maximum comfortable amplitude and at a fixed frequency that matched an audible 0.45 Hz metronome in anterior-posterior (VAP) and medial-lateral (VML) directions, respectively. VAP and VML sway served as a face-validity check of postural sway as a component of the scoring algorithms for the outcome measure of Biosway and SBMA. Subjects were instructed to look straight ahead throughout the trial in eyes open conditions. They were instructed not to bend at the trunk because the thorax segment is modeled kinematically as a rigid body by PiG [21]. Data capture was first started for the MOCAP, and then, the data capture from Biosway and SBMA was started simultaneously by pressing the start button on both instruments and releasing at the same time. A MOCAP-synchronized Bonita video camera (50 Hz) was placed in front of Biosway and SBMA to synchronize MOCAP data with the two instruments. Technical difficulties prevented collection of kinematic data for C7 for one subject, SBMA data for C5–C10 for another subject, and all data for C5–C6 for another two subjects.

### 2.5. Statistical Analyses

Correlation analysis was performed using IBM SPSS Statistics (IBM SPSS Statistics for Windows, Version 23.0. Armonk, NY: IBM Corp.) to examine the linear relationship for all test conditions between (i) SBMA scores and COM Balance indices (COM SI/BOS and COM velocity/BOS), (ii) Biosway SI and COM Balance indices, and (iii) SBMA scores and Biosway SI. Statistical significance was evaluated at *α* = 0.05 with Hochberg adjustments to familywise correlations within each condition [27]. Linearity for correlation analyses were assessed with the Runs test in GraphPad InStat version 3.10 for Windows (GraphPad Software, San Diego California USA), and normality of data sets and residuals were tested with the Shapiro–Wilk test in MedCalc Statistical Software version 17.9.7 (MedCalc Software bvba, Ostend, Belgium). There were no significant deviations from linearity for any correlations (*p* > 0.05), but most of data sets and residuals violated the assumption of normality (*p* < 0.05). For this reason, Kendall's tau-b correlation coefficient (*τ*_b_), a nonparametric measure of association, was used for correlation analysis [28, 29].

One-way repeated measures analysis of variance (ANOVA) was used to assess the effect of condition difficulty on the instruments' balance measures in SPSS. The dependent variables were (i) SBMA scores, (ii) Biosway SI, (iii) Biosway SI/BOS, and (iv) COM Balance indices. The independent variable was the balance protocol with six levels (C1–C8, with scores for conditions C5/C6, and C7/C8 averaged as they represent same level of difficulty of the standing tasks). Most of the data sets violated the assumption of normality; however, due to moderately large sample size, the sampling distribution of the means was assumed to approximate normality based on the central limit theorem [30], so parametric analysis was employed. The assumption of sphericity was tested with Mauchly's test of sphericity, which was significant (*p* < 0.05) for all dependent variables except Biosway SI. Greenhouse–Geisser correction was used for adjustment of degrees of freedom when the assumption of sphericity was violated. Bonferroni post hoc analyses were used to determine pairwise differences between conditions.

## 3. Results

**Figure 2:**
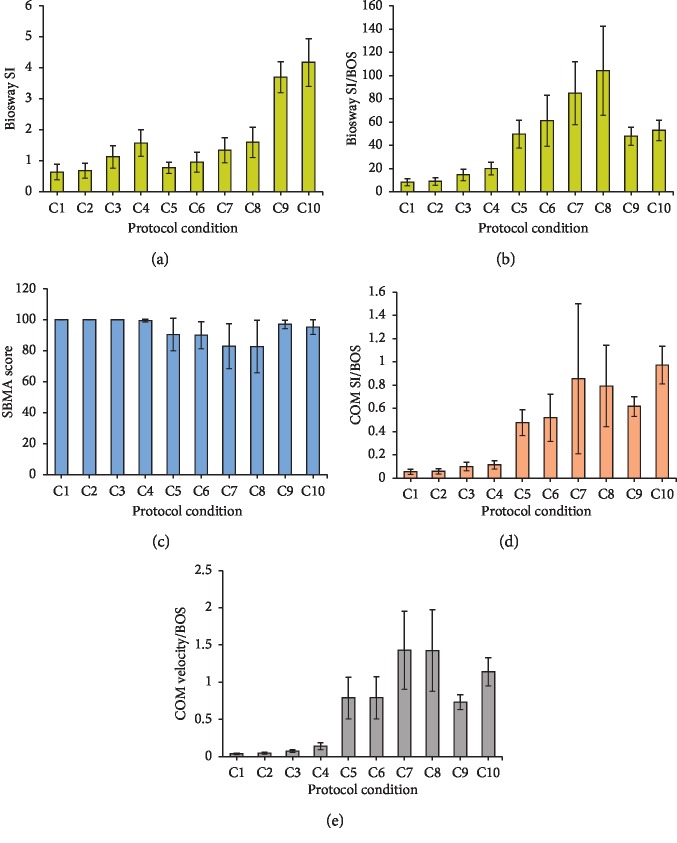
Bar graphs of means ± 1 standard deviation (error bars) for (a) Biosway Sway Index (SI), (b) Biosway SI/Base of Support (BOS), (c) SWAY Balance Mobile Application (SBMA) score, and Center of Mass (COM) Balance indices ((d) COM SI/BOS and (e) COM velocity/BOS) for conditions C1 through C10. A larger velocity and SI score represent more postural sway. A larger SBMA score represents less postural sway.

COM Balance indices (COM SI/BOS and COM velocity/BOS) showed a linear increase in postural sway with increases in condition difficulty level from C1 to C8 (Table 1 and Figures 2(d) and 2(e)). Biosway SI showed a linear increase in postural sway with increases in difficulty level of conditions only for the same type of leg stance; double leg (C1–C4), or single leg (C5–C8) (Table 1 and Figure 2(a)). SBMA scores were near maximal values (100) with very low (zero to near-zero) variance for double leg conditions C1–C4 (Table 1); therefore, statistically valid correlations could not be performed. SBMA scores decreased with increasing condition difficulty only from C5 to C8 (Figure 2(c)). For C9 and C10, both COM Balance indices and Biosway SI showed greater postural sway as the subjects were swaying voluntarily at maximum comfortable amplitude; however, SBMA scores showed less postural sway compared to conditions C5–C8.

The correlations between Biosway SI and both COM Balance indices (COM SI/BOS and COM velocity/BOS) for C1–C8 were significant except between Biosway SI and COM SI/BOS in C5 (Table 2). There were significant correlations between SBMA scores and COM velocity/BOS for conditions C5–C8. The correlations between SBMA scores and COM SI/BOS were significant for conditions C7 and C8 but were not significant for conditions C5 and C6. There were significant correlations between SBMA score and Biosway SI for conditions C5–C8. The correlations between SBMA scores and Biosway SI were nonsignificant for conditions C9 and C10, as were the correlations between SBMA score and COM Balance indices. The correlations between Biosway SI and COM Balance indices were nonsignificant for condition C9 but were significant for C10.

**Figure 3:**
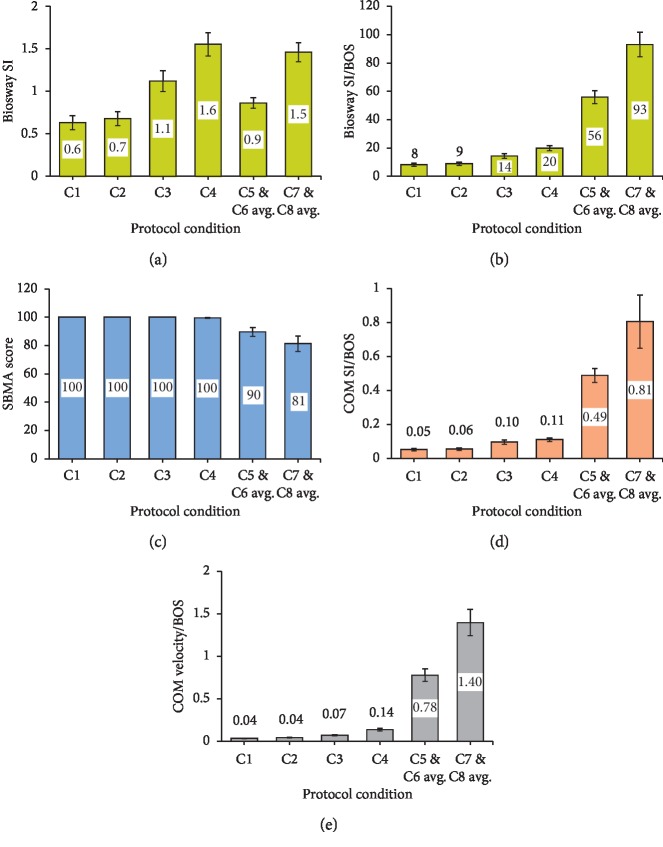
Bar graphs of means with 95% confidence interval (error bars) for dependent variables (a) Biosway Sway Index (SI), (b) Biosway SI/Base of Support (BOS), (c) SWAY Balance Mobile Application (SBMA) score, and Center of Mass (COM) Balance indices ((d) COM SI/BOS and (e) COM velocity/BOS) across increasing level of difficulty (C1–C8) with C5 and C6 averaged and C7 and C8 averaged for analysis of variance (ANOVA). A larger velocity and SI score represent more postural sway. A larger SBMA score represents less postural sway.

There was a significant main effect of the stance conditions for all of the dependent variables (Table 3). Bonferroni post hoc analysis revealed significant differences between conditions C1–C8 for Biosway SI except C1 vs. C2 and C4 vs. C7 and C8 averaged (Figure 3(a)). When BOS was accounted for in Biosway SI, there were significant differences between all conditions except C1 vs. C2 (Figure 3(b)). There were significant differences between all conditions for SWAY score except for C1 vs. C2, C1 vs. C3, and C2 vs. C4 (Figure 3(c)). All pairwise comparisons in ANOVA were significant for COM velocity/BOS across progressively challenging postural stances (C1 to C8) (Figure 3(e)), indicating a valid criterion measure. All pairwise comparisons in ANOVA were significant for COM SI/BOS across conditions C1 to C8 except conditions C1 vs. C2 and C3 vs. C4 (Figure 3(d)), which are eyes open vs. eyes closed conditions.

## 4. Discussion

The current study incorporated a wide range of commonly used clinical standing test conditions for assessing postural stability including double leg stances (mCTSIB), single leg stances, and dynamic voluntary rhythmic sway. The outcome measure from each instrument was correlated during individual stances, thereby eliminating the error in comparing composite scores of test conditions with different stances [31]. The study implemented Vicon's PiG model, the scientific basis of which is previously validated [32, 33], to compute whole body COM position providing a direct measure of balance function. The results of this study indicate that the Biosway is valid for assessment of postural sway in progressively challenging clinical balance protocols; however, a valid comparison of Biosway SI cannot be made across different types of leg stances (double and single leg stance) unless the BOS area is considered. When the BOS area was accounted for in Biosway SI, there was a linear trend with increasing level of difficulty across both types of leg stances (Figure 2(b)) and significant differences between the two types of leg stances (Figure 3(b)). SBMA is valid for assessment of postural sway in a balance assessment protocol consisting of progression from double leg stance to single leg stance and/or progressively challenging single leg stance; however, the ceiling effect during mCTSIB indicates that SBMA lacks sensitivity to detect relatively small postural sway changes in progressively challenging double leg stances in a healthy normal population. Some statistically significant differences in SBMA scores (between C1 and C4 and between C2 and C3) merit evaluation with caution because of the very small effect size; significance was the result primarily due to very small variance in the data samples with values near the ceiling value of 100. Higher SBMA scores for C9 and C10 (Table 1 and Figure 2(c)) indicates that if a subject sways in a uniform rhythmic manner (e.g., Parkinson's disease), the calculation of postural sway based on accelerometers can be inaccurate. Significant correlations between Biosway SI and SBMA scores for C5 to C8 indicate that the scores from either instrument can be used for prediction of scores in the other during progressively challenging single leg stances. COM velocity was more sensitive than COM SI at detecting postural sway changes in conditions with eyes open vs. eyes closed during double leg stances, as COM velocity showed significant differences between these conditions whereas COM SI did not.

A limitation of this study was using a belt to hold the smartphone with SBMA that was tied around the chest at midsternum level, which is different from the SBMA default procedure of holding the device with both hands at the midsternum level. This procedural alteration was necessary to minimize errors in measurement from the movements of device with the participant's hand and to standardize the positioning of the device in all subjects to prevent any inconsistencies due to technique. A limitation of the generalizability of this study included the use of asymptomatic subjects aged 20 to 34 years; therefore, extrapolation to healthy subjects in different age groups or to patient populations with balance impairments may not be appropriate. Areas for further investigations include the assessment of validity of the two instruments in these populations, which can provide more generalizable information about the validity of portable balance assessment instruments that use accelerometers vs. force plates.

## 5. Conclusion

This is the first study to evaluate the concurrent validity of clinical postural sway measurements by accelerometer-based SWAY Balance mobile smartphone application and force plate-based Biodex Biosway using kinematic measures of whole body COM derived from a gold-standard reference, a motion capture system. The study found that Biosway is valid for assessment of postural sway in progressively challenging double leg stances (mCTSIB) and progressively challenging single leg stances, and SBMA is valid for assessment of postural sway in a balance assessment protocol consisting of progression from double leg stance to single leg stance and/or progressively challenging single leg stance. Insights from this study, and further investigations in clinical populations, will be useful in the selection of a portable objective clinical balance assessment instruments.

## Figures and Tables

**Table 1:** Mean ± standard deviation of dependent variables for each test condition.

	C1 Eyes open, firm surface, double leg	C2 Eyes closed, firm surface, double leg	C3 Eyes open, foam surface, double leg	C4 Eyes closed, foam surface, double leg	C5 Eyes open, foam surface, right single leg	C6 Eyes open, foam surface, left single leg	C7 Eyes closed, firm surface, right single leg	C8 Eyes closed, firm surface, left single leg	C9 Eyes open, firm surface, anterior posterior sway	C10 Eyes open, firm surface, medial lateral sway
SBMA score	100.0 ± 0.0	100.0 ± 0.0	100.0 ± 0.0	99.5 ± 0.9	89.9 ± 11.0	89.5 ± 9.4	81.3 ± 18.8	82.1 ± 17.4	96.1 ± 5.4	99.4 ± 6.1
Biosway® SI	0.64 ± 0.24	0.68 ± 0.24	1.15 ± 0.37	1.57 ± 0.42	0.77 ± 0.18	0.96 ± 0.32	1.34 ± 0.39	1.59 ± 0.48	3.68 ± 0.49	4.11 ± 0.80
Mean BOS area (m^2^)	0.077 ± 0.008	0.077 ± 0.080	0.078 ± 0.009	0.079 ± 0.009	0.015 ± 0.004	0.016 ± 0.002	0.016 ± 0.020	0.016 ± 0.002	0.078 ± 0.008	0.079 ± 0.008
Biosway® SI/BOS (m^−2^)	8.23 ± 3.06	8.85 ± 3.26	14.80 ± 5.08	20.08 ± 5.28	50.04 ± 12.07	62.47 ± 23.24	85.58 ± 26.52	103.06 ± 37.38	47.76 ± 7.58	52.45 ± 8.97
COM SI (m)	0.0041 ± 0.0018	0.0043 ± 0.0017	0.0077 ± 0.0027	0.0089 ± 0.0030	0.0073 ± 0.0017	0.0080 ± 0.0029	0.0134 ± 0.0106	0.0123 ± 0.0054	0.0476 ± 0.0070	0.0756 ± 0.0144
COM SI/BOS (m/m^2^)	0.0533 ± 0.0221	0.0556 ± 0.0219	0.0989 ± 0.0362	0.1135 ± 0.0345	0.4722 ± 0.1119	0.5217 ± 0.2018	0.8485 ± 0.6292	0.7879 ± 0.3383	0.6140 ± 0.0881	0.9632 ± 0.1635
COM velocity (m/sec)	0.0028 ± 0.0009	0.0034 ± 0.0010	0.0056 ± 0.0012	0.0107 ± 0.0037	0.0122 ± 0.0041	0.0122 ± 0.0040	0.0225 ± 0.0078	0.0218 ± 0.0074	0.0567 ± 0.0086	0.0883 ± 0.0174
COM velocity/BOS (m^−1^·sec^−1^)	0.036 ± 0.011	0.045 ± 0.013	0.073 ± 0.018	0.137 ± 0.045	0.791 ± 0.278	0.798 ± 0.284	1.44 ± 0.51	1.41 ± 0.53	0.73 ± 0.10	1.13 ± 0.20

Mean ± standard deviation of SWAY Balance Mobile Application (SBMA) scores, Biosway Sway Index (SI), Biosway SI divided by Base of Support (BOS), and Center of Mass (COM) Balance indices (COM SI/BOS and COM velocity/BOS) for conditions C1 through C10. A larger velocity and SI score represent more postural sway. A larger SBMA score represents less postural sway.

**Table 2:** Associations between dependent variables for each test condition.

			Biosway SI	COM SI/BOS	COM velocity/BOS
C1 (eyes open, firm surface, double leg)	Biosway SI	*τ*_b_ value (*N* = 40)	—	**0.818**	**0.411**
		*p* value	—	**<0.0001**	**<0.001**
C2 (eyes closed, firm surface, double leg)	Biosway SI	*τ*_b_ value (*N* = 40)	—	**0.751**	**0.355**
		*p* value	—	**<0.0001**	**<0.01**
C3 (eyes open, foam surface, double leg)	Biosway SI	*τ*_b_ value (*N* = 40)	—	**0.781**	**0.382**
		*p* value	—	**<0.0001**	**<0.001**
C4 (eyes closed, foam surface, double leg)	Biosway SI	*τ*_b_ value (*N* = 40)	—	**0.590**	**0.433**
		*p* value	—	**<0.0001**	**<0.0001**
C5 (eyes open, foam surface, right single leg)	SBMA score	*τ*_b_ value (*N* = 37)	**−0.353**	−0.086	**−0.534**
		*p* value	**<0.01**	0.456	**<0.0001**
	Biosway SI	*τ*_b_ value (*N* = 38)	—	0.209	**0.304**
		*p* value	—	0.068	**<0.01**
C6 (eyes open, foam surface, left single leg)	SBMA score	*τ*_b_ value (*N* = 37)	**−0.441**	−0.170	**−0.545**
		*p* value	**<0.001**	0.139	**<0.0001**
	Biosway SI	*τ*_b_ value (*N* = 38)	—	**0.409**	**0.575**
		*p* value	—	**<0.001**	**<0.0001**
C7 (eyes closed, firm surface, right single leg)	SBMA score	*τ*_b_ value (*N* = 38)	**−0.439**	**−0.319**	**−0.632**
		*p* value	**<0.001**	**<0.01**	**<0.0001**
	Biosway SI	*τ*_b_ value (*N* = 39)	—	**0.325**	**0.469**
		*p* value	—	**<0.01**	**<0.0001**
C8 (eyes closed, firm surface, left single leg)	SBMA score	*τ*_b_ value (*N* = 39)	**−0.305**	**−0.384**	**−0.570**
		*p* value	**<0.01**	**<0.01**	**<0.0001**
	Biosway SI	*τ*_b_ value (*N* = 40)	—	**0.302**	**0.483**
		*p* value	—	**<0.01**	**<0.0001**
C9 (eyes open, firm surface, anterior posterior sway)	SBMA score	*τ*_b_ value (*N* = 39)	0.070	0.099	0.131
		*p* value	0.529	0.377	0.241
	Biosway SI	*τ*_b_ value (*N* = 40)	—	−0.027	0.119
		*p* value	—	0.807	0.278
C10 (eyes open, firm surface, medial lateral sway)	SBMA score	*τ*_b_ value (*N* = 39)	0.087	0.138	0.192
		*p* value	0.439	0.217	0.086
	Biosway SI	*τ*_b_ value (*N* = 40)	—	**0.249**	**0.341**
		*p* value	—	**<0.05**	**<0.01**

Kendall's tau-b correlation coefficient (*τ*_b_) and *p* value with Hochberg *α* adjustments (significant correlations are presented in bold) for correlations between SWAY Balance Mobile Application (SBMA) scores and Biosway Sway Index (SI), SBMA scores and Center of Mass (COM) Balance indices (COM SI/Base of Support (BOS) and COM velocity/BOS), and Biosway SI and COM Balance indices. A larger velocity and SI score represent more postural sway. A larger SBMA score represents less postural sway.

**Table 3:** Summary table of one-way repeated measure analysis of variance (ANOVA) for dependent variables.

Variable	df		*F*	Sig.	Partial eta squared (*η*^2^)	Observed power
	Between groups	Within groups				
Biosway SI	5	185	70.63	*p* < 0.001	0.656	1.00
SBMA score	1.31	47.25	44.78	*p* < 0.001	0.554	1.00
Biosway SI/BOS	1.70	63.05	309.5	*p* < 0.001	0.893	1.00
COM SI/BOS	1.86	68.86	51.78	*p* < 0.001	0.583	1.00
COM velocity/BOS	1.37	50.58	300.9	*p* < 0.001	0.890	1.00

Main effect of condition difficulty for dependent variables Biosway Sway Index (SI), SWAY Balance Mobile Application (SBMA) score, Biosway SI/Base of Support (BOS), and Center of Mass (COM) Balance indices (COM SI/BOS and COM velocity/BOS) with Greenhouse–Geisser corrected degrees of freedom except for Biosway SI.

## Data Availability

The data used to support the findings of this study are available from the corresponding author upon request.
